# Betabloqueadores para Hipertensão: Ser ou Não Ser um Medicamento de Primeira Linha?

**DOI:** 10.36660/abc.20250684

**Published:** 2026-02-06

**Authors:** José Augusto Soares Barreto-Filho, Felipe Vieira Santana, Thiago Augusto da Silva Nascimento, Pedro Gabriel Melo de Barros e Silva

**Affiliations:** 1 Universidade Federal de Sergipe Aracaju SE Brasil Universidade Federal de Sergipe, Aracaju, SE – Brasil; 2 Hospital São Lucas Rede D’Or São Luiz Aracaju SE Brasil Hospital São Lucas, Rede D’Or São Luiz, Aracaju, SE – Brasil; 3 Hospital do Coração São Paulo SP Brasil Hospital do Coração (Hcor), São Paulo, SP – Brasil; 4 Universidade Federal de São Paulo São Paulo SP Brasil Universidade Federal de São Paulo, São Paulo, SP – Brasil; 5 Brazilian Clinical Research Institute São Paulo SP Brasil Brazilian Clinical Research Institute (BCRI), São Paulo, SP – Brasil

**Keywords:** Hipertensão, Betabloqueadores

O Prêmio Nobel Sir James Black^1^ foi pioneiro no conceito e design de betabloqueadores para uso cardiovascular. Os primeiros dados humanos publicados sobre betabloqueadores surgiram em 1964 e, desde então, os betabloqueadores têm sido amplamente utilizados em diversas condições cardiovasculares. Na hipertensão, o estudo pivotal do *Medical Research Council* (MRC) foi o primeiro a demonstrar que tanto o propranolol quanto a bendrofluazida reduziram o risco de acidente vascular cerebral em pacientes com hipertensão leve; no entanto, nenhuma redução significativa na mortalidade foi observada.^2^ Análises comparativas entre propranolol e bendrofluazida mostraram resultados semelhantes, com uma tendência não significativa favorecendo a bendrofluazida na prevenção de acidente vascular cerebral e o propranolol na redução de eventos coronários em não fumantes.^2^

Historicamente, os betabloqueadores se tornaram um pilar no tratamento da hipertensão. Em 1984, o Comitê Nacional Conjunto para Detecção, Avaliação e Tratamento da Hipertensão Arterial (JNC III) classificou os betabloqueadores, juntamente com os diuréticos tiazídicos, como terapia de primeira linha.^3^ Em 1998, Messerli et al. publicaram uma revisão sistemática questionando a adequação dos betabloqueadores como agentes de primeira linha, concluindo que, em pacientes idosos hipertensos, os betabloqueadores eram inferiores aos diuréticos na redução dos desfechos cardiovasculares.^4^

Em 2003, o JNC-7, amplamente influenciado pelos resultados do estudo ALLHAT, estabeleceu os diuréticos tiazídicos como a pedra angular do tratamento da hipertensão.^5^ Desde então, inúmeras meta-análises e consensos de especialistas reforçaram as preocupações sobre a eficácia comparativa dos betabloqueadores na prevenção de desfechos cardiovasculares, particularmente quando comparados a diuréticos tiazídicos, inibidores da enzima conversora de angiotensina (IECA), bloqueadores dos receptores da angiotensina (BRAs) e bloqueadores dos canais de cálcio (BCCs).^6–8^ Esse crescente conjunto de evidências levou muitas sociedades cardiovasculares a rebaixar os betabloqueadores para terapia de segunda linha para hipertensão não complicada.^9,10^

Mais recentemente, no entanto, a Sociedade Europeia de Hipertensão (ESH), em suas diretrizes de 2023, reintegrou os betabloqueadores entre as cinco principais classes de medicamentos para o tratamento da hipertensão.^11^ Estas incluem IECA, BRA, betabloqueadores, BCC e diuréticos tiazídicos/semelhantes aos tiazídicos. A maioria das diretrizes enfatiza o uso de betabloqueadores quando há indicações convincentes, como angina, insuficiência cardíaca ou arritmia. Essa posição reacendeu o debate entre especialistas em hipertensão.^11^

Para subsidiar o desenvolvimento da atualização das Diretrizes Brasileiras de Hipertensão, a Sociedade Brasileira de Cardiologia encomendou revisão sistemática sobre a eficácia do atenolol para hipertensão, publicada nesta edição dos Arquivos Brasileiros de Cardiologia (ABC Cardiol). Brandão et al.^12^ merecem elogios por sua análise rigorosa e oportuna, com o objetivo de gerar Recomendação Clínica focada nesta questão PICO específica. Esta iniciativa está alinhada ao compromisso da Sociedade Brasileira de Cardiologia em aprimorar a robustez metodológica de futuras atualizações das diretrizes.

Os autores seguiram um protocolo sistemático para identificar ensaios clínicos randomizados comparando o atenolol com outros agentes anti-hipertensivos de primeira linha. O atenolol foi selecionado devido à sua inclusão na Relação Nacional de Medicamentos Essenciais do Brasil (RENAME 2024) e no Programa Farmácia Popular. Os estudos incluídos foram revisões sistemáticas de ECRs ou ECRs originais com pelo menos dois braços comparativos. O desfecho primário foi um composto de eventos cardiovasculares maiores, compreendendo mortalidade por todas as causas, acidente vascular cerebral e infarto do miocárdio; e os desfechos secundários analisando esses eventos individualmente. É importante ressaltar que tanto a significância estatística quanto as diferenças clinicamente relevantes mínimas foram consideradas. O risco de viés foi avaliado utlizando-se a ferramenta RoB 2, e a certeza da evidência foi classificada usando a estrutura GRADE. Meta-análises de comparações diretas entre o atenolol e outros agentes anti-hipertensivos foram apresentadas, com a revisão de Wiysonge et al. (2017) servindo como fonte primária.^13^

Com base nesses resultados, a Recomendação Clínica foi desenvolvida por um painel de especialistas nomeado pela Sociedade Brasileira de Cardiologia. A revisão sistemática e o processo de recomendação foram conduzidos de forma independente por metodologistas, sem conflitos de interesse relatados. Esse esforço reflete um compromisso mais amplo com a tomada de decisões baseada em evidências, tanto no cenário clínico quanto nas políticas de saúde pública.

Apesar da qualidade e transparência do processo, persistem diversas limitações metodológicas inerentes aos estudos originais, tornando a recomendação mais uma questão hermenêutica do que uma decisão firmemente baseada em evidências. A imprecisão das evidências disponíveis, refletida no risco de viés avaliado e na certeza geral dos estudos que as sustentam, deve ser reconhecida. Uma perspectiva holística é essencial, especialmente considerando que a hipertensão continua sendo o principal fator de risco modificável para doenças cardiovasculares em todo o mundo.

Esta revisão destaca uma lacuna crítica: a ausência de ensaios clínicos comparativos de alta qualidade, tanto históricos quanto contemporâneos, comparando os betabloqueadores a outros agentes anti-hipertensivos. Por exemplo, as evidências fundamentais provêm, em grande parte, de estudos publicados entre 1986 e 2005, conforme resumido na meta-análise de Wiysonge et al.^13^ Isso limita a aplicabilidade dos resultados ao contexto clínico atual. Pode-se argumentar que betabloqueadores de terceira geração poderiam levar a melhores resultados, mas isso não parece ser o caso.^14^

Várias questões-chave surgem para ajudar na interpretação desses dados e construção da recomendação:

1. **Metas de Pressão Arterial podem ser mais Importantes do que a Classe de Medicamentos**: Alcançar o controle adequado da pressão arterial, especialmente em pacientes mais jovens, pode ser mais importante do que a escolha de classes específicas de medicamentos. Os betabloqueadores continuam eficazes na redução da pressão arterial, principalmente em adultos mais jovens.
2. **A Terapia Combinada é a Norma**: Na prática clínica atual, a maioria dos pacientes inicia o tratamento com terapia combinada. Assim, os efeitos pleiotrópicos de medicamentos individuais podem ser menos relevantes do que as estratégias gerais de controle da PA.
3. **Imprecisão da Eficácia Comparativa**: Muitos ensaios clínicos incluídos apresentaram imprecisão crítica na estimativa da superioridade de uma classe sobre outra. Em alguns casos, as comparações foram injustas — por exemplo, atenolol versus uma combinação de hidroclorotiazida e amilorida.
4. **Novas Evidências do Ensaio Clínico QUARTET**: O estudo QUARTET demonstrou que iniciar a terapia com uma *quadpill* de baixa dose, incluindo bisoprolol, foi superior à monoterapia, reforçando a relevância dos betabloqueadores em regimes de combinação.^15^
5. **Proteção Diferencial de Órgãos-alvo**: Diferentes classes de medicamentos podem conferir efeitos protetores distintos em órgãos-alvo afetados pela hipertensão, complicando ainda mais as classificações hierárquicas de eficácia.
6. **Comorbidades Influenciam a Escolha do Medicamento**: Em cenários do mundo real, os betabloqueadores são frequentemente indicados para condições concomitantes, como arritmia, angina ou insuficiência cardíaca, reforçando ainda mais seu papel no tratamento da hipertensão.
7. **Custo-efetividade e Acessibilidade**: Em um sistema de saúde universal como o brasileiro, a acessibilidade e a disponibilidade de betabloqueadores devem ser levadas em consideração nas decisões de prescrição.

Levando em conta todas essas considerações, concordamos com a conclusão dos autores. A modesta, e potencialmente discutível, inferioridade dos betabloqueadores em certos desfechos cardiovasculares, em comparação com o chamado "Trio Dourado" (diuréticos tiazídicos, IECA/BRA e BCC), não é sustentada por evidências contemporâneas sólidas. Em vez de foco na restrição do uso de betabloqueadores, as estratégias clínicas devem priorizar a obtenção do controle ideal da pressão arterial desde o diagnóstico da hipertensão, idealmente antes do início da lesão subclínica em órgãos-alvo. Assim, em geral, argumentamos contra a restrição das opções terapêuticas disponíveis para o manejo da hipertensão. Apesar das potenciais vantagens de outras classes de medicamentos anti-hipertensivos, uma estratégia mais impactante seria democratizar o acesso a todas as classes de medicamentos eficazes, incluindo betabloqueadores, para maximizar o controle da pressão arterial com terapias combinadas e, em última análise, alcançarmos a almejada redução do risco cardiovascular em nível populacional.
