# Supplementary material for: Betabloqueadores para Hipertensão: Ser ou Não Ser um Medicamento de Primeira Linha?
Source: Arq Bras Cardiol. 2026 Feb 6;122(9):e20250684. [Article in Portuguese] doi: 10.36660/abc.20250684 (PMC12978169; doi:10.36660/abc.20250684)
Supplement: Translation

# Translation: Beta-blockers for Hypertension: To Be or Not to Be a First-line Drug?

**DOI:** 10.36660/abc.20250684i

**Authors:** José Augusto Soares Barreto-Filho, Felipe Vieira Santana, Thiago Augusto da Silva Nascimento, Pedro Gabriel Melo de Barros e Silva

**Affiliations:** 1 Aracaju SE Brazil Universidade Federal de Sergipe, Aracaju, SE – Brazil; 2 Aracaju SE Brazil Hospital São Lucas, Rede D’Or São Luiz, Aracaju, SE – Brazil; 3 São Paulo SP Brazil Hospital do Coração (Hcor), São Paulo, SP – Brazil; 4 São Paulo SP Brazil Universidade Federal de São Paulo, São Paulo, SP – Brazil; 5 São Paulo SP Brazil Brazilian Clinical Research Institute (BCRI), São Paulo, SP – Brazil

**Keywords:** Hypertension, Beta-blockers

Nobel laureate Sir James Black^1^ pioneered the concept and design of beta-blockers for cardiovascular use. The first human data published on beta-blockers appeared in 1964, and ever since, beta-blockers have been widely utilized across various cardiovascular conditions. In hypertension, the landmark Medical Research Council (MRC) trial was the first to demonstrate that either propranolol or bendrofluazide reduced the risk of stroke in patients with mild hypertension; however, no significant reduction in mortality was observed.^2^ Comparative analyses between propranolol and bendrofluazide showed similar outcomes, with a non-significant trend favoring bendrofluazide for stroke prevention and propranolol for coronary event reduction in non-smokers.^2^

Historically, beta-blockers became a mainstay in hypertension management. In 1984, the Joint National Committee on Detection, Evaluation, and Treatment of High Blood Pressure (JNC III) classified beta-blockers alongside thiazide diuretics as first-line therapy.^3^ In 1998, Messerli et al. published a systematic review questioning the appropriateness of beta-blockers as first-line agents, concluding that in elderly hypertensive patients, beta-blockers were inferior to diuretics in reducing cardiovascular outcomes.^4^

In 2003, the JNC-7, largely influenced by results from the ALLHAT trial, established thiazide diuretics as the cornerstone of hypertension treatment.^5^ Since then, numerous meta-analyses and expert consensus statements have reinforced concerns about the comparative efficacy of beta-blockers in preventing cardiovascular outcomes, particularly when compared to thiazide diuretics, angiotensin-converting enzyme inhibitors (ACEi), angiotensin receptor blockers (ARBs), and calcium channel blockers (CCBs).^6–8^ This growing body of evidence led many cardiovascular societies to downgrade beta-blockers to second-line therapy for uncomplicated hypertension.^9,10^

More recently, however, the European Society of Hypertension (ESH), in its 2023 guidelines, reinstated beta-blockers among the top five drug classes for managing hypertension.^11^ These include ACEi, ARBs, beta-blockers, CCBs, and thiazide/thiazide-like diuretics. Most of the guidelines emphasize the use of beta-blockers when compelling indications are present, such as angina, heart failure, or arrhythmia. This position has reignited debate among hypertension experts.^11^

To support the development of an updated edition of the Brazilian Guidelines for Hypertension, the *Sociedade Brasileira de Cardiologia* commissioned a systematic review on the efficacy of atenolol for hypertension, published in this issue of the *Arquivos Brasileiros de Cardiologia* (ABC Cardiol). Brandão et al.^12^ are to be commended for their rigorous and timely analysis aimed at generating a focused Clinical Recommendation based on this specific PICO question. This initiative aligns with the *Sociedade Brasileira de Cardiologia*'s commitment to enhancing the methodological robustness of future guideline updates.

The authors followed a systematic protocol to identify randomized clinical trials comparing atenolol with other first-line antihypertensive agents. Atenolol was selected due to its inclusion in Brazil's National List of Essential Medicines (RENAME 2024) and the Popular Pharmacy Program. Included studies were either systematic reviews of RCTs or original RCTs with at least two comparative arms. The primary outcome was a composite of major cardiovascular events, comprising all-cause mortality, stroke, and myocardial infarction, with secondary outcomes analyzing these events individually. Importantly, both statistical significance and minimal clinically relevant differences were considered. Risk of bias was assessed using the RoB 2 tool, and the certainty of the evidence was graded using the GRADE framework. Meta-analyses of direct comparisons between atenolol and other antihypertensive agents were presented, with the review by Wiysonge et al. (2017) serving as the primary source.^13^

Based on these results, the Clinical Recommendation was developed by an expert panel appointed by the *Sociedade Brasileira de Cardiologia*. The systematic review and recommendation process were conducted independently by methodologists with no reported conflicts of interest. This effort reflects a broader commitment to evidence-based decision-making in both clinical scenario and public health policy.

Despite the quality and transparency of the process, several methodological limitations inherent to the original studies persist, rendering the recommendation more of a hermeneutic issue than a firmly evidence-based decision. The imprecision in the available evidence, reflected in the assessed risk of bias and the overall certainty of supporting studies, must be acknowledged. A holistic perspective is essential, particularly given that hypertension remains the leading modifiable risk factor for cardiovascular disease worldwide.

This review highlights a critical gap: the absence of both historical and contemporary high-quality head-to-head trials comparing beta-blockers to other anti-hypertensive agents. For instance, the foundational evidence stems largely from studies published between 1986 and 2005, as summarized in Wiysonge et al.'s meta-analysis.^13^ This limits the applicability of the findings to today's clinical context. One may argue that third-generation beta-blockers could lead to better results, but this does not seem to be the case.^14^

Several key issues emerge to help the interpretation of these data for building the recommendation:

1. **BP Targets May Matter More Than Drug Class**: Achieving adequate blood pressure control, especially in younger patients, might outweigh the choice of specific drug classes. Beta-blockers remain effective at lowering BP, particularly in younger adults.
2. **Combination Therapy is the Norm**: In current clinical practice, most patients begin treatment with combination therapy. Thus, the pleiotropic effects of individual drugs may be less relevant than overall BP control strategies.
3. **Imprecision of Comparative Effectiveness**: Many included trials exhibited a critical imprecision in estimating the superiority of one class over another. In some cases, comparisons involved unfair matchups—e.g., atenolol versus a combination of hydrochlorothiazide and amiloride.
4. **Emerging Evidence from the QUARTET Trial**: The QUARTET trial showed that initiating therapy with a low-dose quadpill, including bisoprolol, was superior to monotherapy, reinforcing the relevance of beta-blockers within combination regimens.^15^
5. **Differential Target Organ Protection**: Different drug classes may confer distinct protective effects on target organs affected by hypertension, further complicating hierarchical classifications of efficacy.
6. **Comorbidities Influence Drug Choice**: In real-world settings, beta-blockers are frequently indicated for concomitant conditions such as arrhythmia, angina, or heart failure, further supporting their continued role in hypertension management.
7. **Cost-effectiveness and Accessibility**: In a universal health system like Brazil's, the affordability and availability of beta-blockers should be factored into prescribing decisions.

Taking together all these considerations, we agree with the authors’ conclusion. The modest, and potentially debatable, inferiority of beta-blockers in certain cardiovascular outcomes, compared to the so-called "Golden Trio" (thiazide diuretics, ACEi/ARBs, and CCBs), is not supported by strong contemporary evidence. Rather than focusing on restricting beta-blocker use, clinical strategies should prioritize achieving optimal blood pressure control from the point of hypertension diagnosis, ideally before the onset of subclinical target organ damage. Thus, in general, we argue against narrowing the therapeutic options available for managing hypertension. Despite the potential advantages of other classes of antihypertensive medications, a more impactful strategy would be to democratize access to all effective medication classes, including beta-blockers, to maximize blood pressure control with combination therapies and ultimately achieve the cardiovascular risk reduction at the population level.
